# Supplementary material for: Differential functions of FANCI and FANCD2 ubiquitination stabilize ID2 complex on DNA
Source: EMBO Rep. 2020 Jun 8;21(7):e50133. doi: 10.15252/embr.202050133 (PMC7332966; doi:10.15252/embr.202050133)
Supplement: Supplementary file 1 — Appendix [file EMBR-21-e50133-s001.pdf]

Table of Contents

APPENDIX TABLE S1 2

APPENDIX TABLE S2 2

Appendix Table S1. DNA substrates

Name	Sequence (5' to 3')
ds32	CGATCGGTAACGTATGCTGAATCTGGTGCTGG<br>CCAGCACCAGATTCAGCATACGTTACCGATCG
ds32[F]	/5IRD700/CGATCGGTAACGTATGCTGAATCTGGTGCTGG<br>/5IRD700/CCAGCACCAGATTCAGCATACGTTACCGATCG
ds50	CGTCGACTCTACATGAAGCTCGAAGCCATGAATTCAAATGACCTCTGATCA<br>TGATCAGAGGTCATTTGAATTCATGGCTTCGAGCTTCATGTAGAGTCGACG
ss64	TTTCCCAGCACCAGATTCAGCATACGTTACCGATCGTACGTTCGATGCTGGCTACTGCTAGCTT

Appendix Table S2. CryoEM data collection

	$I_{Ub}D2_{Ub}$-dsDNA	$ID2_{Ub}$
Microscope	Titan Krios	Cryoarm
Detector	Falcon III	DE64
Magnification	80,000x	200,000x
Voltage (kV)	300	300
Electron Dose ($e^-/Å^2$)	44.4	46.2
Defocus range (µm)	1.0-3.5	1.0-3.5
Pixel Size (Å)	1.085	0.598
