# Supplementary material for: Differential functions of FANCI and FANCD2 ubiquitination stabilize ID2 complex on DNA
Source: EMBO Rep. 2020 Jun 8;21(7):e50133. doi: 10.15252/embr.202050133 (PMC7332966; doi:10.15252/embr.202050133)
Supplement: Supplementary file 2 — Expanded View Figures PDF [file EMBR-21-e50133-s002.pdf]

Expanded View Figures

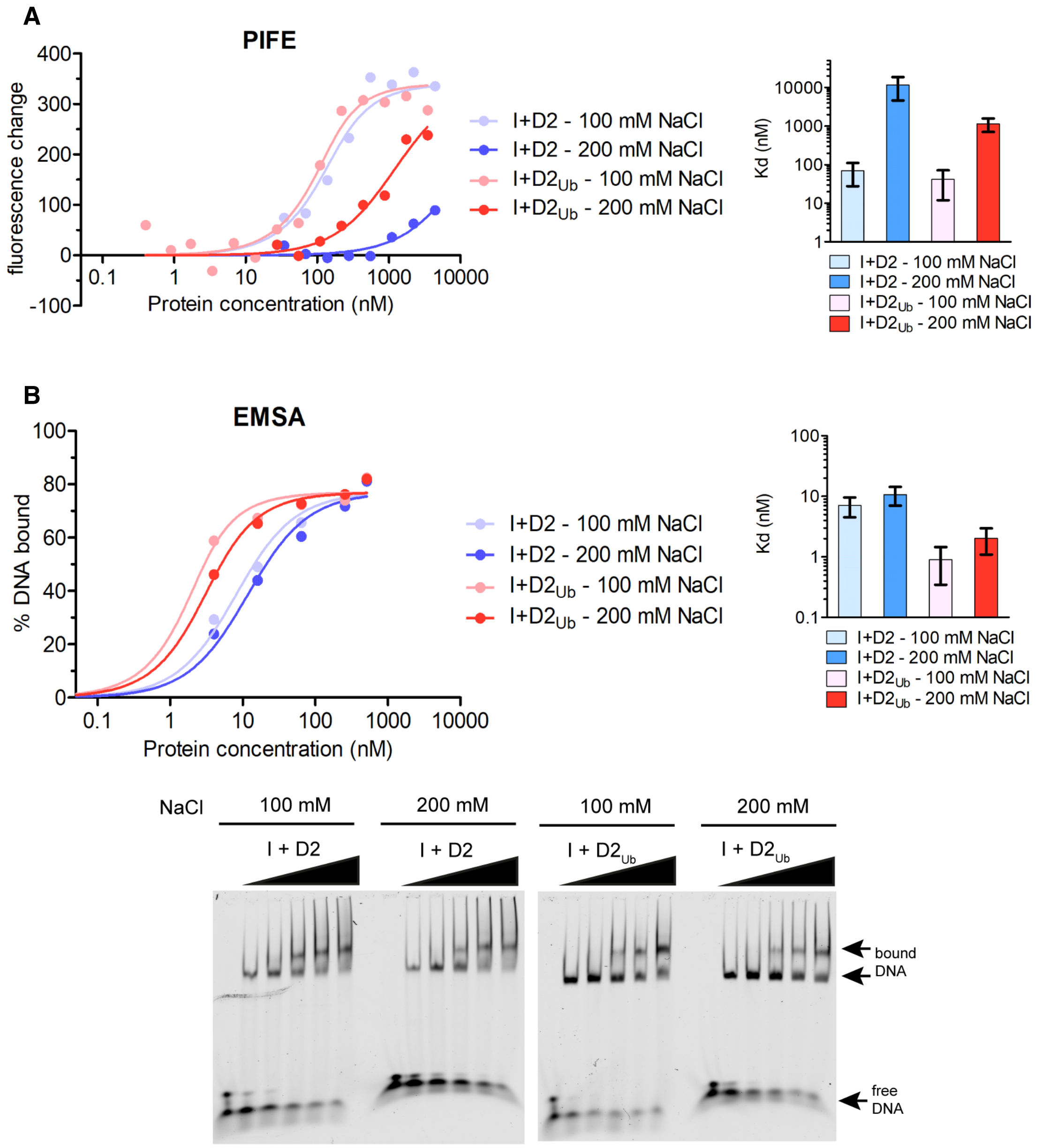

Figure EV1. PIFE and EMSA salt dependence on ID2-dsDNA and ID2$_{Ub}$-dsDNA interactions.

A, B ID2-dsDNA and ID2$_{Ub}$-dsDNA binding assays at 100 and 200 mM NaCl, using either PIFE (A) or EMSA (B). IRDye700-labelled dsDNA (125 nM for PIFE or 2 nM for EMSA) was incubated with increasing concentrations of ID2 or ID2$_{Ub}$, and % DNA binding and fluorescence changes were determined by EMSA and PIFE, respectively. Corresponding values for each concentration were used in fitting of a one-site binding model. Mean apparent K_d values calculated for each complex are shown on the left. Error bars: Asymmetric 95% confidence intervals from non-linear regression (5–14 data points each).

Source data are available online for this figure.

© 2020 The Authors

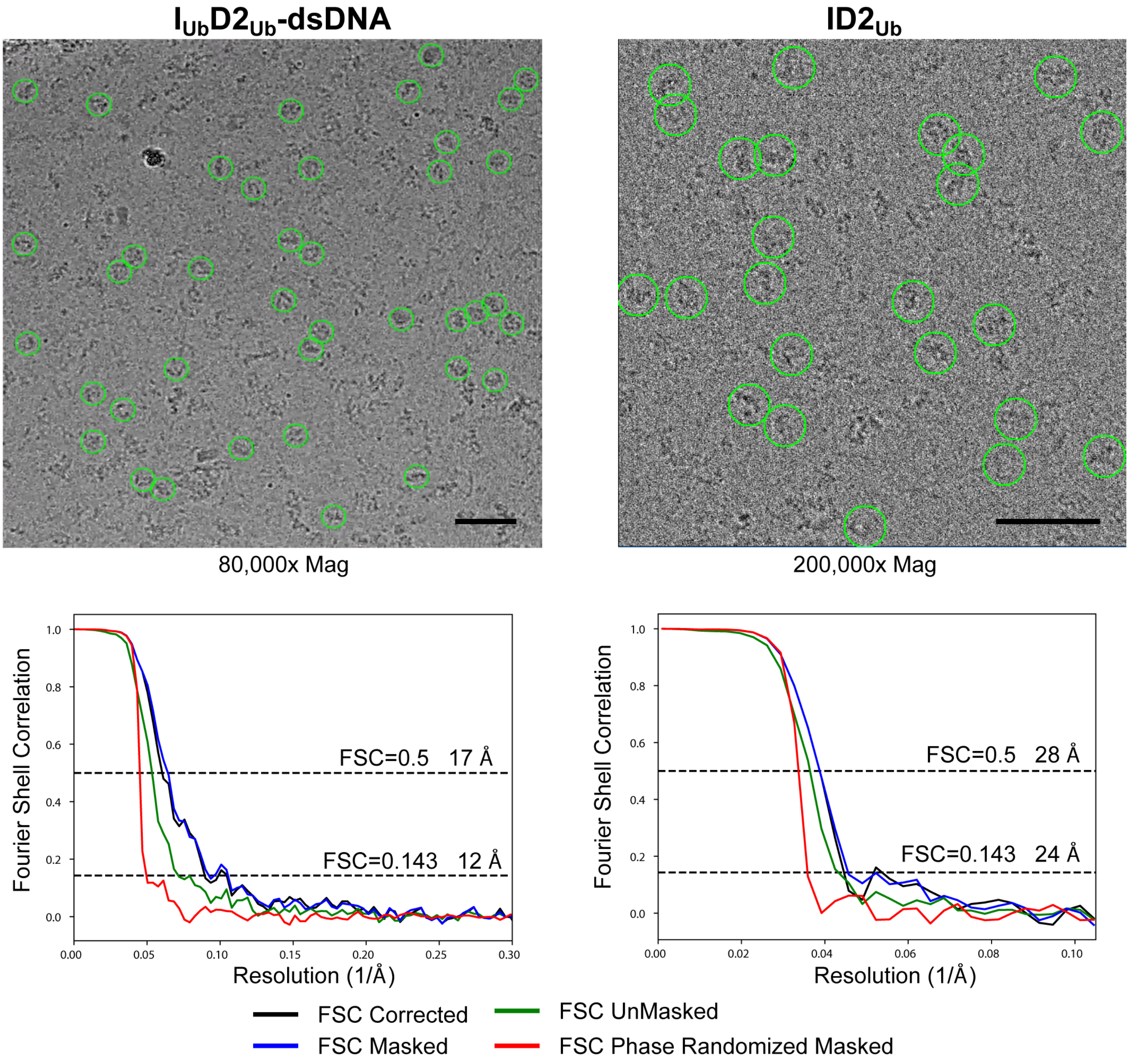

Figure EV2. CryoEM data analysis for $I_{Ub}D2_{Ub}$-dsDNA and $ID2_{Ub}$ samples.

Top: Selected micrographs with some particles indicated with green circles. Scale bars are 50 nm. *Bottom*: Fourier shell correlation (FCS) curves for each dataset. Masking was performed with phase randomization to 23 Å ($I_{Ub}D2_{Ub}$-dsDNA) and 31 Å ($ID2_{Ub}$), and the indicated resolutions are for the corrected FSC curves.

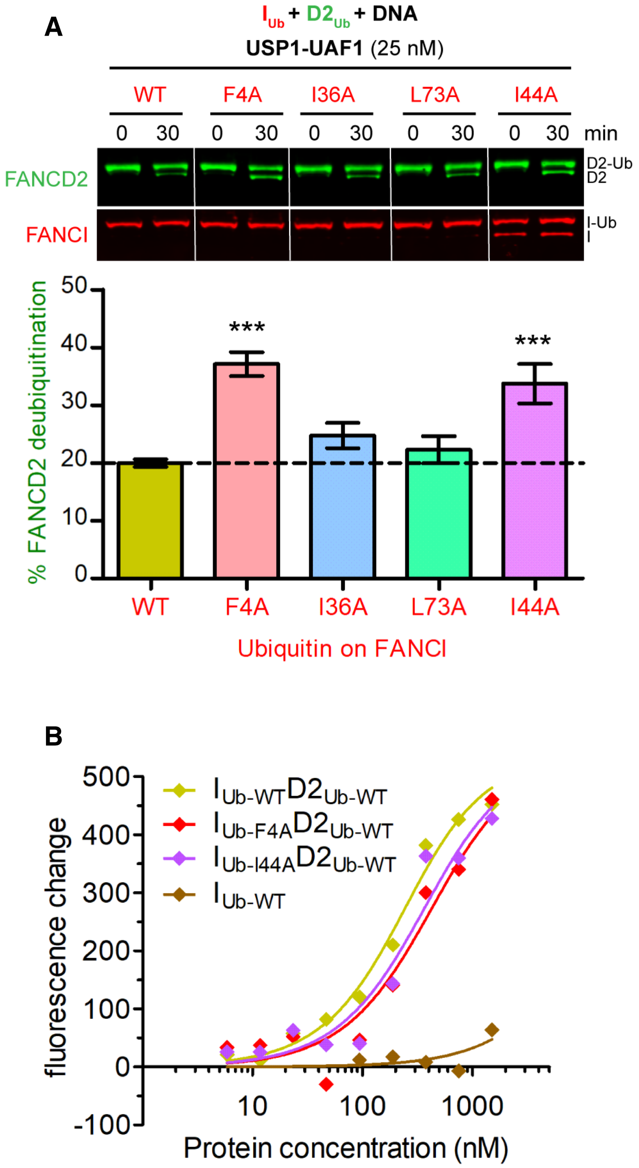

Figure EV3. F4 and I44 in FANCI's conjugated ubiquitin are required for efficient protection of FANCD2's ubiquitin from USP1-UAF1-mediated deubiquitination.

A FANCI was first ubiquitinated *in vitro* in the presence of excess dsDNA, using wild-type ubiquitin (WT) or indicated ubiquitin mutants. Ubiquitinated FANCI products were then mixed with ubiquitinated FANCD2, and resulting $I_{Ub}D2_{Ub}$-DNA complexes were incubated with USP1-UAF1 (25 nM) for 30 min. Deubiquitination of FANCD2 and FANCI at 0 and 30 min was monitored by Western blotting. The % FANCD2 deubiquitination over this period, calculated from FANCD2 blots deriving from three replicate DUB experiments, was plotted for each ubiquitin type (mean ± SD). Statistically significant changes compared to WT ubiquitin (one-way ANOVA test with Bonferroni correction) are indicated with asterisks. ***$P < 0.001$.

B $I_{Ub}D2_{Ub}$ complexes with F4A or I44A ubiquitin mutants on FANCI can efficiently associate with dsDNA. PIFE DNA binding curves for $I_{Ub}D2_{Ub}$ complexes consisting of $D2_{Ub}$, and $I_{Ub\text{-}mut}$ produced with either wild-type (WT) ubiquitin, or indicated ubiquitin mutants. IRDye700-labelled dsDNA (at 125 nM) was incubated at increasing concentrations of corresponding $I_{Ub}D2_{Ub}$ complexes or I_{Ub} control (ranging from 5.86 nM to 1.5 μM), and recorded fluorescence changes were plotted for each protein concentration along with the fit of a one-site binding model.

Source data are available online for this figure.

 © 2020 The Authors

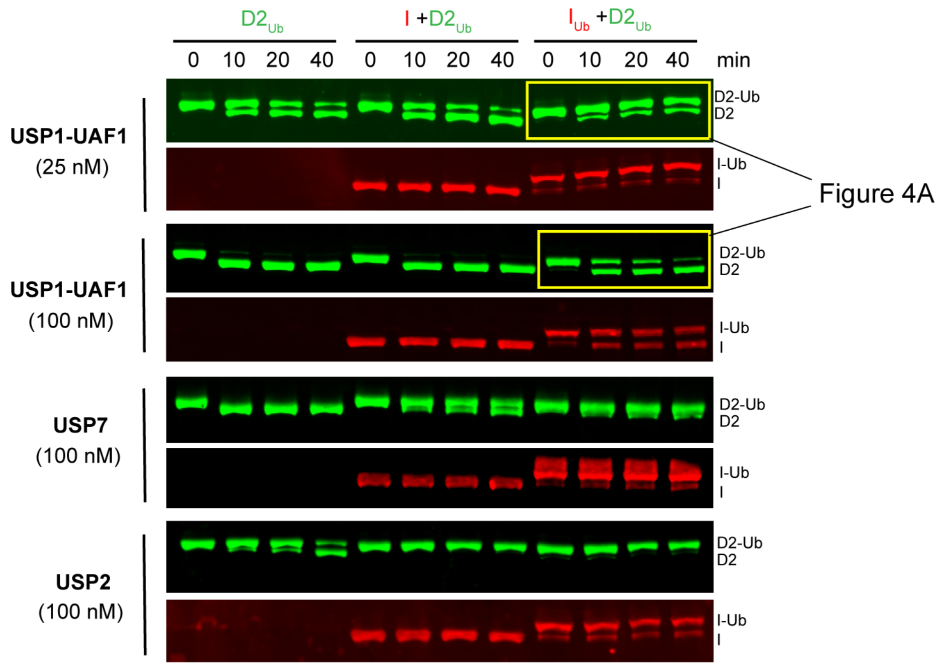

Figure EV4. ID2$_{Ub}$ complex is resistant to general DUB activity, whereas I$_{Ub}$D2$_{Ub}$ is additionally resistant to USP1-UAF1 activity.

Ubiquitinated FANCD2 (D2$_{Ub}$) was mixed with a 50 base pair dsDNA and with either His$_6$-TEV-V5-FANCI (I), ubiquitinated His$_6$-TEV-V5-FANCI (I$_{Ub}$) or no protein; protein–DNA mixes were subsequently incubated with either USP1-UAF1 (at 25 nM or 100 nM), USP7 (100 nM) or USP2 (100 nM), for indicated time periods. Deubiquitination of D2$_{Ub}$ and I$_{Ub}$ was assessed, following SDS–PAGE, by Western blotting of transferred blots with specific FANCD2 and V5/FANCI antibodies.

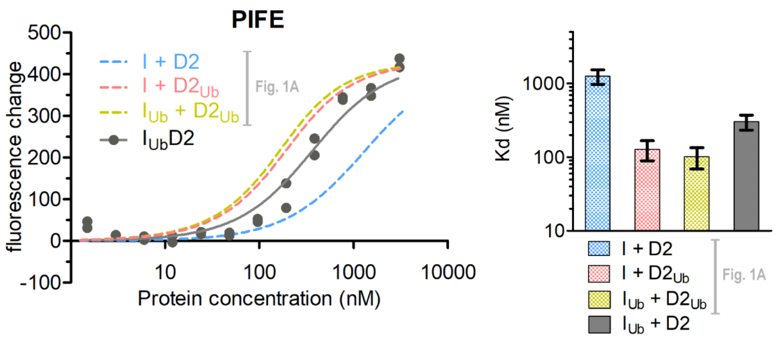

Figure EV5. I$_{Ub}$D2 complex has an intermediate affinity for dsDNA, relative to ID2 and ID2$_{Ub}$.

Left: Fluorescence changes of IRDye700-labelled dsDNA (at 125 nM) when incubated at increasing I$_{Ub}$D2 concentrations (ranging from 1.7 nM to 2.9 μM). Measurement of fluorescence enhancement was conducted for two separately prepared complexes (two technical replicates), and all data points were used in fitting of a one-site binding model. *Right*: Bar graph showing mean apparent K_d values calculated from the one-site binding model. Error bars: Asymmetric 95% confidence intervals from non-linear regression (24 data points each). I + D2, I + D2$_{Ub}$ and I$_{Ub}$ + D2$_{Ub}$ previously calculated curves and corresponding K_d values (from data points shown in Fig 1A) are also shown for comparison.

Source data are available online for this figure.

© 2020 The Authors
